# Comparison of immune cell profiles associated with heatstroke, sepsis, or cardiopulmonary bypass: Study protocol for an exploratory, case-control study trial

**DOI:** 10.3389/fmed.2023.1165786

**Published:** 2023-04-17

**Authors:** Juan Wu, Sha Yang, Tingting Wang, Qinjuan Wu, Xinyi Liao, Rong Yao, Lei Du

**Affiliations:** ^1^Department of Anesthesiology, West China Hospital, Sichuan University, Chengdu, China; ^2^Department of Emergency Medicine, West China Hospital, Sichuan University, Chengdu, China; ^3^Department of Anesthesiology, Second Affiliated Hospital, Zhejiang University School of Medicine, Hangzhou, China

**Keywords:** heatstroke, sepsis, cardiopulmonary bypass, immune profile, flow cytometry

## Abstract

**Introduction:**

Heatstroke is a life-threatening illness involving extreme hyperthermia and multi-organ failure, and it is associated with high mortality. The immune profiles of heatstroke have not been fully elucidated, and diagnostic and prognostic biomarkers of heatstroke are lacking. This study will analyze immune profiles in heatstroke patients as they differ from profiles in patients with sepsis or aseptic inflammation patients in order to identify diagnostic and prognostic biomarkers.

**Methods:**

This exploratory, case–control study will recruit patients with heatstroke, patients with sepsis, patients undergoing cardiopulmonary bypass as well as healthy controls at West China Hospital of Sichuan University from 1 January 2023 to 31 October 2023. The four cohorts will be profiled at one time point in terms of lymphocytes, monocytes, natural killer cells, and granulocytes using flow cytometry, and cell populations will be visualized in two dimensions using t-SNE and UMAP, then clustered using PhenoGraph and FlowSOM. Gene expression in the specific immune cell populations will also be compared across the four cohorts, as will levels of plasma cytokines using enzyme-linked immunosorbent assays. Outcomes in the cohorts will be monitored during 30-day follow-up.

**Discussion:**

This trial is, to our knowledge, the first attempt to improve the diagnosis of heatstroke and prediction of prognosis based on immune cell profiles. The study is also likely to generate new insights into immune responses during heatstroke, which may help clarify the disease process and lay the foundation for immunotherapies.

## 1. Introduction

Heatstroke, which can be categorized as classic heatstroke (CHS) or exertional heatstroke (EHS) depending on its cause (1), is the most life-threatening form of hyperthermia (>40.5°C), and it involves central nervous system dysfunction and multi-organ failure (2). Even with intensive care, mortality rates of heatstroke patients can be as high as 30–60% (1, 2). The incidence of heatstroke-related deaths has been increasing globally during the past 2 decades, as record-breaking heatwaves have occurred (1). Heatstroke survivors may experience long-term neurological and cardiovascular complications (1, 2).

Hyperthermia can damage proteins, lipids, and nucleic acids, as well as kill cells directly (3, 4), inducing the production of cytokines to protect against tissue injury (5, 6). If thermoregulation fails to maintain core temperature within an appropriate range, inflammatory responses may become excessive and injure tissue, leading to multi-organ failure and death (7–9), akin to systemic inflammatory response syndrome (SIRS) (10–15). In addition, heatstroke can compromise gastrointestinal integrity, allowing endotoxins and pathogens to enter the circulation and trigger endotoxemia (14). However, potentially informative differences in immune profiles among patients with heatstroke, SIRS, or sepsis have not been fully elucidated.

For lack of a specific biomarker (1), heatstroke is diagnosed based on hyperthermia, history of exposure to extreme heat or vigorous exertion, and alterations of the central nervous system (6, 7). Researchers have undertaken genomic, epigenomic, transcriptomic, proteomic, and metabolomic studies in an effort to identify diagnostic biomarkers (16–18), without much success. The rate of false positive diagnosis of heatstroke remains as high as 24% and the rate of false negative diagnosis as high as 64.5%, especially among patients with atypical symptoms (2). To make matters worse, heatstroke can be difficult to differentiate from sepsis, especially when it involves leukocytosis, which can delay cooling treatments that substantially improve prognosis (1).

Therefore, the aims of this case–control study are to identify the specific immune cells biomarker(s) to diagnose heatstroke and predict prognosis by exploring the immune profile of heatstroke as it differs from profiles in aseptic inflammation and sepsis. The results will help us more comprehensively understand the immune system characteristics of heatstroke, improve its diagnosis, and provide a basis for the development of immunotherapy. In the trial, patients with heatstroke, patients with sepsis, patients undergoing cardiopulmonary bypass [CPB, representative of aseptic inflammation (19)], and healthy controls will be compared in terms of their profiles of lymphocytes, monocytes, natural killer cells, and granulocytes in circulation, based on flow cytometry; in terms of gene expression in different immune cell populations, based on single-cell RNA sequencing; and in terms of cytokine levels in plasma, based on enzyme-linked immunosorbent assay (ELISA). The three cohorts of patients will be matched according to age, sex, weight, and comorbidities. Healthy controls will be matched to heatstroke patients based on age, sex and weight.

## 2. Methods and analysis

**Figure 1:**
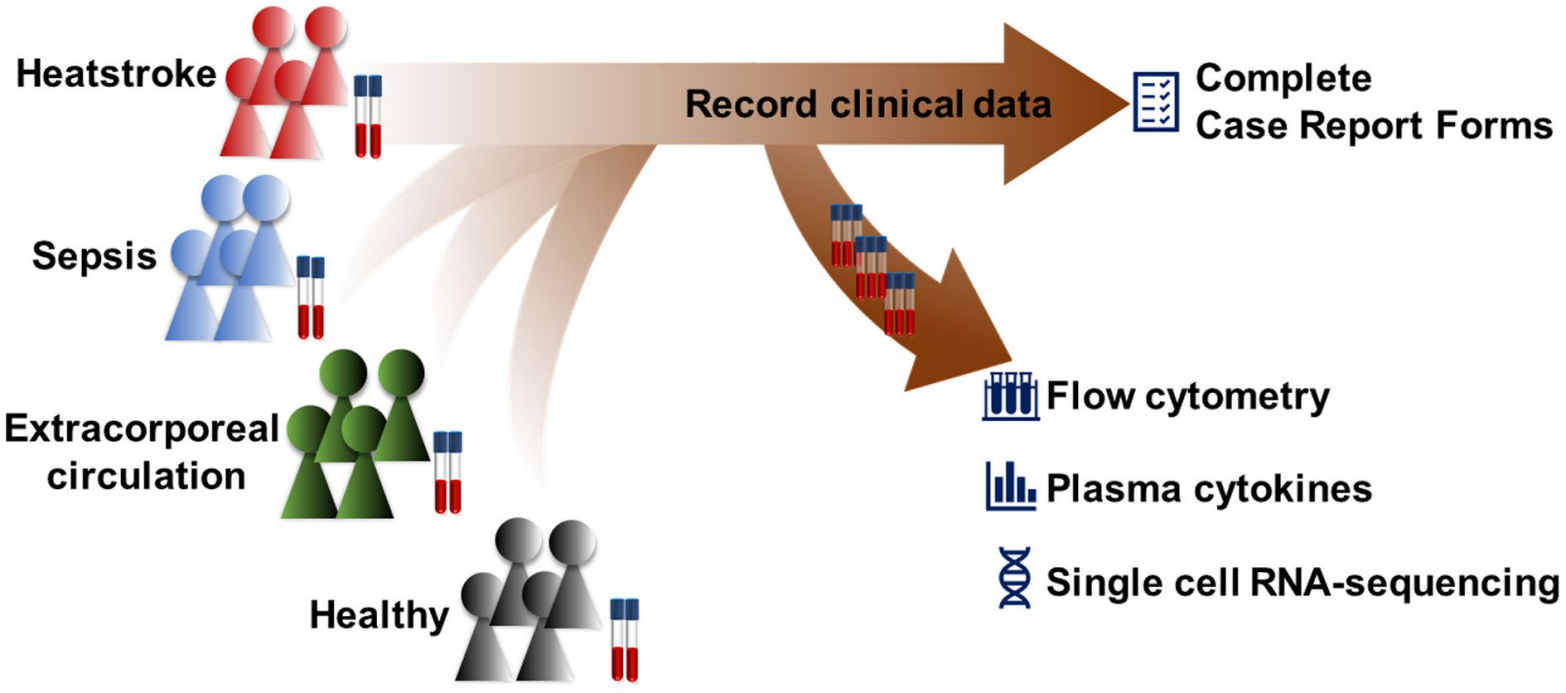
Presentation of the study design.

The protocol of this exploratory, case–control study has been approved by the Medical Ethics Committee of West China Hospital, Sichuan University (2022–1408), and it has been registered at the Chinese Clinical Trial Registry^1^ under ChiCTR-2200066952. This protocol follows the SPIRIT guidelines, and the SPIRIT checklist was provided in Supplementary Table 1. The study will be coordinated by the International Medical Center and by the Departments of Anesthesiology and of Emergency Medicine at West China Hospital of Sichuan University (Figure 1).

### 2.1. Participant selection and matching

#### 2.1.1. Inclusion and exclusion criteria

Participants will be recruited from the International Medical Center and from the Departments of Emergency Medicine and of Anesthesiology at West China Hospital of Sichuan University. Four cohorts of 40 individuals each will be recruited: heatstroke patients, sepsis patients, patients undergoing CPB, and healthy controls. The inclusion and exclusion criteria for each cohort are shown in Table 1. Participant recruitment and data collection will begin on January 1, 2023 and will continue until October 31, 2023.

**Table 1:** Inclusion and exclusion criteria for subject enrollment.

Subjects	Inclusion criteria	Exclusion criteria
Heatstroke patients	(1) Age ≥ 18 years	(1) Pregnant women.
	(2) History of exposure to extreme environmental heat or vigorous muscle exertion	
	(3) Core body temperature > 40°C or symptoms of central nervous system (CNS) dysfunction, such as coma, delirium, or convulsions	(2) Comorbid with immune system disease within the 6 months prior to admission, including but not limited to acquired immune deficiency syndrome (AIDS), multiple myeloma, rheumatoid arthritis, systemic lupus erythematosus, multiple sclerosis, myasthenia gravis, scleroderma, Crohn’s disease, and ulcerative colitis.
	(4) Written informed consent to participate	
Sepsis patients	(1) Age ≥ 18 years	(3) Comorbid with malignant tumor and hematological diseases.
	(2) Suspected or known infection	
	(3) Sequential organ failure assessment (SOFA) score ≥ 2	
	(4) Written informed consent to participate	(4) History of organ or bone marrow transplantation.
Patients undergoing CPB	(1) Age ≥ 18 years	
	(2) Valve replacement or coronary artery bypass under cardiopulmonary bypass	
	(3) Written informed consent to participate	
Healthy controls	(1) Age ≥ 18 years	(5) History of immunosuppressive therapy, including glucocorticoids, soluble microbial products (SMSs), polyclonal and monoclonal antibodies, antimetabolite and alkylating agents, or radiotherapy within the 6 months prior to admission.
	(2) Body mass index 18–24 kg/m^2^	
	(3) Health examination within the previous 6 months indicated no abnormality	
	(4) Written informed consent to participate	

#### 2.1.2. Participant matching

Heatstroke patients will be matched 1:1 with sepsis patients, CPB patients and healthy controls using propensity score analysis based on nearest-neighbor matching and a caliper width of 0.2 of the standard deviation of the logit of the propensity score (20). Covariates during matching of heatstroke and sepsis patients will be sex, age, body weight, Sequential Organ Failure Assessment (SOFA) score (21), comorbidities, and treatment history. Covariates during matching of heatstroke and CPB patients will be sex, age, body weight, comorbidities, and treatment history. Covariates during matching of heatstroke patients and healthy controls will be sex, age, and body weight.

### 2.2. Endpoints

The aims of this case–control study are to identify the diagnostic immune cells biomarker(s) of heatstroke and prognostic immune cells biomarker(s) of heatstroke within 30 days.

For exploring diagnostic biomarker(s) that distinguishes patients with heatstroke from patients with sepsis, patients with aseptic inflammation and healthy individuals, because this trial is a case–control study, no endpoints can be provided.

For exploring prognostic biomarker(s) for patients with heatstroke, the primary endpoint is all-cause mortality within 30 days. The secondary endpoints include incidence of the organ injury events within 30 days, including neurological dysfunction, acute heart failure, acute renal failure, or respiratory failure, which are diagnosed as following:

Neurological dysfunction will include stroke, which will be diagnosed based on symptoms of unconsciousness or focal neurologic deficit and the results of computerized tomography and magnetic resonance imaging; and delirium, which will be diagnosed as described (22).

Acute heart failure will be diagnosed according to symptoms of breathlessness, tiredness, and fatigue; clinical signs; abnormalities of electrocardiogram(ECG) such as atrial fibrillation, Q waves, left ventricle hypertrophy, and a widened QRS complex (23); left ventricular ejection fraction (LVEF) ≤ 40% based on the results of echocardiography (23); and elevated levels of B-type natriuretic peptide (BNP) or NT-proBNP (24).

Acute renal failure will be defined as stage 3 acute kidney injury according to the KDIGO criteria (25): stage3: creatinine level in serum ≥4.0 mg/dL (353.60 μmol/L) or ≥ 3 times baseline within 7 days, or requirement for renal replacement therapy, or urine output <0.3 mL/kg/h for ≥24 h OR anuria for ≥12 h.

Respiratory failure will be defined as pH < 7.35 and PaCO_2_ > 45 mmHg or as PaO_2_/FiO_2_ < 200 based on invasive or non-invasive ventilation or arterial blood gas analysis (26, 27) within 30 days. If patients with concomitant pulmonary diseases prior to admission meet above diagnostic criteria, the patient will not be considered to have lung injury caused by heatstroke and will be excluded.

### 2.3. Minimal sample size

We did not calculate minimal sample size to achieve a given statistical power because this is an exploratory study (28, 29) and we are unaware of other studies with a similar design and purpose. Recent studies of the protective effects of precooling and curcumin on organ injury among patients with EHS included only 8–12 participants (30, 31). A study that examined immune cell dynamics during EHS was able to obtain statistically significant results using only 11 patients (32). Therefore, to differentiate the immune profiles, we aim to enroll at least 40 subjects in each of the four cohorts.

### 2.4. Study protocol

Specially trained investigators will screen individuals for potential inclusion based on the inclusion and exclusion criteria, and they will obtain written informed consent from the individuals or their legal representatives. The items to be measured are listed in Table 2. The following baseline data will be recorded from all participants on case report forms: age, sex, ethnicity, profession, education level, body mass index, history of smoking and drinking, family medical history, laboratory results, comorbidities, and treatments. Comorbidities include cardiovascular disease, hypertension, asthma, chronic obstructive pulmonary disease, epilepsy, stroke, diabetes, hyperthyroidism, hypothyroidism, chronic liver disease, virus infection, and neurological disease. Treatments include antihypertensive agents, aspirin, statins, anticoagulant drug, oral antidiabetic agents, insulin, and levothyroxine.

**Table 2:** Schedule of enrollment, intervention, and assessment.

Items	Screening	Post-allocation	1 month after trial
	Visit1 (Within 24 h of admission)	Visit2 (During hospitalization)	Visit3
Informed consent	**×**		
Inclusion/exclusion criteria	**×**		
Basic information^a^	**×**		
Smoking and drinking history	**×**		
Family medical history	**×**		
Comorbidities and treatments^b^	**×**		
The location of onset, symptoms and vital signs on admission (for heatstroke patients and sepsis patients)	**×**		
Preoperative data (for CPB patients)		**×**	
Intraoperative data (for CPB patients)		**×**	
Mechanical ventilation		**×**	
Renal replacement therapy		**×**	
Transfusion		**×**	
Usage of medication^c^		**×**	
Death			**×**
Infection			**×**
Complications^d^			**×**

a Age, gender, job, height, and weight, etc.

b Comorbidities include cardiovascular disease, hypertension, asthma, chronic obstructive pulmonary disease, epilepsy, stroke, diabetes, hyperthyroidism, hypothyroidism, chronic liver disease, virus infection, and neurological disease. Treatments include antihypertensive agents, aspirin, statins, anticoagulant drug, oral antidiabetic agents, insulin, and euthyrox.

c Including vasoactive agents, glucocorticoids, and antibiotics.

d Including cardiovascular, respiratory, nervous, digestive or urinary systems complications.

Additional information will be recorded onto case report forms for the three patient cohorts. In the case of heatstroke and sepsis patients, the location of onset, symptoms, and vital signs on admission will be collected. In the case of CPB patients, preoperative data will be recorded on surgical diagnosis, New York Heart Association cardiac function classification, and EuroSCORE II, as will intraoperative data about surgical procedure, surgery time, anesthesia time, amount of blood transfusion, urine output, and medicines used during surgery. We will record mechanical ventilation, renal replacement therapy, and transfusion, as well as use of vasoactive agents, glucocorticoids, and antibiotics during hospitalization for the three patient cohorts. These groups will be followed up at 30 days after admission, either in person if the individual remains in hospital or by telephone or WeChat if the individual has been discharged. During follow-up, data will be collected on death, infection, and complications affecting cardiovascular, respiratory, nervous, digestive, or urinary systems.

These data will be entered onto case report forms. To protect confidentiality, the files will be stored in a secure and locked place and manner, and the subject identification and private information will be deleted from all study documents. To control the quality of data, supervising investigators will be required to ensure the accuracy and completeness of the data. Then the data will be doubly entered into the EpiData 3.10 database system by two investigators. After finishing the data entry and dealing with the query, the database will be locked under the orders of the principal investigator. No one is allowed to view the database without authorization; otherwise, they should notify the principal investigator.

#### 2.4.1. Blood collection

Venous blood (10 mL) will be collected into tubes coated with ethylenediamine tetraacetic acid within 24 h after admission in the case of heatstroke and sepsis patients, at the end of CPB in the case of CPB patients, and during outpatient visits at the International Medical Center in the case of healthy controls.

Within 4 h after collection, 8 mL of the blood samples will be processed to isolate peripheral blood mononuclear cells (PBMCs; see section 2.4.2), while the remaining 2 mL of blood will be centrifuged at 100 *g* at 4°C for 5 min, and then 500 μL of plasma will be stored at −80°C for cytokine assays (see section 2.4.5).

#### 2.4.2. Preparation of PBMCs

Venous blood obtained as described in section 2.4.1 will be added to 10 volumes of NH_4_Cl solution and allowed to sit at room temperature for 10 min to lyse the erythrocytes. Samples will be centrifuged at 400 *g* at 4°C for 10 min, the supernatant will be discarded, and the pellet will be resuspended in phosphate-buffered saline (PBS) containing 2% fetal bovine serum. The resuspension will be centrifuged at 400 *g* at 4°C for 10 min again, and the pelleted PBMCs will be resuspended in PBS to a concentration of 2 × 10^6^/mL based on cell counting (33).

#### 2.4.3. Flow cytometry to determine immune cell profiles

Aliquots of PBMCs (100 μL) will be stained with appropriate antibodies for 30 min at room temperature in the dark in order to label, in separate samples, T lymphocytes, B lymphocytes, monocytes, NK cells, and granulocytes (Table 3). Then samples will be centrifuged at 300 *g* at 4°C for 5 min, the supernatant will be discarded, the pellet will be resuspended in 300 μL PBS, and the suspension will be measured using a FACSLyric ™ flow cytometer (BD Bioscience). The technician performing flow cytometry will be blinded to the origin of the samples.

**Table 3:** Antibody panels to identify immune cell subsets by flow cytometry.

Fluorophore	Antigen	Fluorophore	Antigen
T cell panel 1		T cell panel 2	
BV421	CD279	BV421	CD279
BV510	CD25	BV510	CD8
BV605	CD45	BV605	CD45
FITC	CCR7	FITC	CD38
PE	CD127	PE	CD69
PerCP-Cy5.5	CD3	PerCP-Cy5.5	CD3
PE-Cy7	CD28	PE-Cy7	CD28
APC	CD45RA	APC	CD45RO
APC-R700	FVS	APC-R700	FVS
APC-Cy7	CD4	APC-Cy7	CD4
B cell panel		Natural killer cell panel	
BV421	CD27	BV421	CD57
BV510	CD19	BV510	CD335
BV605	CD45	BV605	CD45
FITC	CD38	FITC	Lin(CD19/CD14/CD123/CD11C/FC)
PE	IgG	PE	CD127
PerCP-Cy5.5	IgM	PerCP-Cy5.5	CD3
PE-Cy7	CD274	PE-Cy7	CD56
APC	CD80	APC	CD314
APC-R700	FVS	APC-R700	FVS
APC-Cy7	IgD	APC-Cy7	CD16
Monocyte panel		Granulocyte panel	
BV421	CD284	BV421	CD123
BV510	CD40	BV510	CD15
BV605	CD45	BV605	CD45
FITC	CD14	FITC	CD14
PE	HLA-DR	PE	Siglec8
PerCP-Cy5.5	CD80	PerCP-Cy5.5	CD54
PE-Cy7	CD274	PE-Cy7	CD11b
APC	CD163	APC	CD181
APC-R700	FVS	APC-R700	FVS
APC-Cy7	CD16	APC-Cy7	CD64
		BV786	CD16

Flow cytometry data will be analyzed using FlowJo software (Treestar, Ashland, OR, United States), which will be gated to exclude debris, dead cells and doublets but retain single live cells for subsequent analysis. Immune cell subsets will be visualized in two dimensions map using t-distributed stochastic neighbor embedding (t-SNE) (34) and the “uniform manifold approximation and projection” algorithm (UMAP) (35). Then cells will be grouped into phenotypically homogeneous clusters using PhenoGraph (36) or FlowSOM (37), and the clusters will be characterized using Marker Enrichment Modeling (38).

#### 2.4.4. Assay of cytokine levels in plasma

Plasma samples will be analyzed for the following cytokines using commercial ELISAs: perforin, granzyme B, granulocyte-macrophage colony-stimulating factor, high mobility group box-1, heat shock protein-72, tumor necrosis factor-α, interferon-γ as well as interleukins (ILs)-1α, 1β, 2, 4, 6, 10, and 21, which will be measured according to the manufacturer’s instructions. Calibration curves will also be prepared according to the manufacturer’s instructions. Absorbance at appropriate wavelengths will be measured using a microplate reader (39).

#### 2.4.5. Single-cell RNA sequencing

Peripheral blood mononuclear cells will be prepared as described in section 2.4.2, and their viability will be assessed using trypan blue staining. When cell viability exceeds 85%, 5 × 10^4^ cells will be captured and used to construct libraries with the Chromium Next GEM Single Cell 5’ Reagent Kit (10x Genomics) according to the manufacturer’s instructions. The cells will be loaded into each channel, then partitioned into gel bead emulsions in the Chromium instrument, then followed as described (40).

### 2.5. Statistical analysis

Statistical analyses will be performed using Prism version 9 (GraphPad, San Diego, CA, United States). All statistical tests will be two-tailed, and results associated with *p* < 0.05 will be considered significant.

Continuous variables will be checked for normal distribution using the Shapiro–Wilk test and D’Agostino-Pearson omnibus normality test. If normally distributed, continuous data will be reported as mean (SD), and inter-cohort differences will be assessed for significance using one-way ANOVA or Student’s *t*-test. If skewed, continuous data will be reported as median (range or interquartile range), and inter-cohort differences and multiple pairwise comparisons will be assessed using a non-parametric Kruskal Wallis test or Mann–Whitney test.

Categorical variables will be reported as the number of cases (percentage). Inter-cohort differences in such variables will be assessed using the Chi-squared test or Fisher’s exact test. Inter-cohort differences in ordinal variables will be assessed using the Kruskal Wallis test or Mann–Whitney test comparisons.

Subgroup analyses will be performed according to the type of heatstroke (CHS or EHS). CHS always results from passive exposure to high ambient temperature, often accompanied by high humidity. EHS, in contrast, occurs in young individuals during vigorous exercise in hot or temperate environments (1).

Student’s *t*-test or Mann–Whitney test will be used to detect correlations between specific immune cell subsets and prognosis of heatstroke patients. The ability of such subsets to predict heatstroke and prognosis of affected individuals will be assessed using receiver operating characteristic curves, based on optimal thresholds obtained by maximizing the Youden index. Sensitivity, specificity, predictive values, and likelihood ratios will be calculated.

## 3. Discussion

This trial aims to be the first systematic, comprehensive comparison of immune cell profiles across three conditions that can be difficult to differentiate clinically: heatstroke, aseptic inflammation such as in patients undergoing CPB (19), and systemic infection such as in sepsis patients. Our study may succeed in identifying specific immune cell signatures useful for accurate diagnosis of heatstroke and for identifying patients at high risk of worse outcomes. Regardless of whether the study achieves this goal, it should provide a comprehensive understanding of the immune profiles in heatstroke, which may help clarify the course of this life-threatening condition and lay the foundations for immunotherapies that might become the first curative treatments for heatstroke.

To reduce the potential bias of clinicodemographic differences among the four cohorts on their immune cell profiles, we will match the groups based on age, sex, and body mass index.

## Ethics statement

The studies involving human participants were reviewed and approved by the Medical Ethics Committee of West China Hospital, Sichuan University. The patients/participants provided their written informed consent to participate in this study.

## Author contributions

LD and RY designed the study. JW, SY, TW, QW, and XL performed the experiments and analyzed the data. JW drafted this protocol. All authors contributed to the article and approved the submitted version.

## Funding

This work is supported by the 1•3•5 Outstanding Development Program of West China Hospital of Sichuan University (2017-120).

## Conflict of interest

The authors declare that the research was conducted in the absence of any commercial or financial relationships that could be construed as a potential conflict of interest.

## Publisher’s note

All claims expressed in this article are solely those of the authors and do not necessarily represent those of their affiliated organizations, or those of the publisher, the editors and the reviewers. Any product that may be evaluated in this article, or claim that may be made by its manufacturer, is not guaranteed or endorsed by the publisher.

## Footnotes

^1^ www.chictr.org.cn
